# Impacts of ambient air pollution on glucose metabolism in Korean adults: a Korea National Health and Nutrition Examination Survey study

**DOI:** 10.1186/s12940-020-00623-9

**Published:** 2020-06-17

**Authors:** Myung-Jae Hwang, Jong-Hun Kim, Youn-Seo Koo, Hui-Young Yun, Hae-Kwan Cheong

**Affiliations:** 1 Department of Social and Preventive Medicine, Sungkyunkwan University School of Medicine, 2066 Seobu-ro Jangan-gu, Suwon, Gyeonggi-do 16419 Republic of Korea; 2 Department of Environmental and Energy Engineering, Anyang University, Anyang, South Korea

**Keywords:** Air pollution, Korea National Health and nutritional examination survey, Fasting blood sugar, Hemoglobin A1c, Diabetes

## Abstract

**Background:**

Exposure to air pollution was reported to affect glucose metabolism, increasing the risk of diabetes mellitus. We conducted an epidemiological study on glucose metabolism and air pollution by exploring the levels of fasting blood glucose (FBG) and hemoglobin A1c (HbA1c) with changes in ambient air quality, depending on the characteristics of the susceptible population.

**Methods:**

We carried out a cross-sectional analysis of a nationally representative sample of 10,014 adults (4267 in male and 5747 in female) from the Korea National Health and Nutrition Examination Survey in 2012 and 2013 along with data from the Korean Air Quality Forecasting System. The analysis was performed using a generalized linear model stratified by sex, age, and presence of diabetes. We assessed the changes in FBG and HbA1c associated with exposures to particulate matter (PM_10_), fine particulate matter (PM_2.5_), and nitrogen dioxide (NO_2_) after controlling for confounders.

**Results:**

There were 1110 participants with diabetes (557 in male and 553 in female). Overall, the FBG level increased by 7.83 mg/dL (95% confidence interval [CI]: 2.80–12.87) per interquartile range (IQR) increment of NO_2_, 5.32 mg/dL (95% CI: 1.22–9.41) per IQR increment of PM_10_ at a moving average of 0–6 days, and 4.69 mg/dL (95% CI: 0.48–8.91) per IQR increment of PM_2.5_ at a moving average of 0–5 days. HbA1c increased by 0.57% (95% CI: 0.04–1.09) per IQR increment of PM_10_ at a moving average of 0–60 days and 0.34% (95% CI: 0.04–0.63) per IQR increment of PM_2.5_ at a moving average of 0–75 days. The change in FBG and HbA1c increased more in the diabetic group, especially in males aged 65 years or more. There was a strong association between elevation in diabetes-related parameters and exposure to air pollution.

**Conclusions:**

Our study provides scientific evidence supporting that short- and mid-term exposure to air pollution is associated with changes in biological markers related to diabetes. This finding suggests that the impact of air pollution should be reflected in chronic disease management when establishing local health care policies.

## Background

Exposure to ambient air pollution is a major environmental risk factor in terms of the global burden of disease [1]. Short- and long-term exposure to particulate matter (PM) increases the incidence of respiratory and cardiovascular disease [2], as well as the risk of chronic diseases such as diabetes mellitus (DM) [3, 4]. DM is a disease in which insulin secretion is perturbed, resulting in impaired blood glucose control. Exposure to ambient air pollutants has also been associated with deterioration in blood coagulation and glucose metabolism and effects on gene expression [5–8]. These metabolic disorders are associated with an increase in the levels of fasting blood glucose (FBG) and hemoglobin A1c (HbA1c), which are essential blood markers of DM.

Prolonged inhalation of toxic substances in the atmosphere leads to an increased risk of DM [9–15]. Exposure to nitrogen dioxide (NO_2_) contributes to impaired glucose metabolism [16], while exposure to fine particulate matter (PM_2.5_) resulted in elevated levels of FBG and HbA1c [17, 18]. Chen et al. observed a strong association between daily exposure to air pollutants and changes in FBG levels [19]. Chuang et al. also reported that short-term exposure to PM_10_ elevated the levels of FBG and HbA1c [20].

However, air pollution does not affect all populations equally because of the differences in sensitivity among susceptible subpopulation [21]. Several previous studies have assessed the effects of short-term exposure to ambient air pollution on the morbidity associated with DM, obesity, and hypertension in susceptible populations [22–24]. In 2012, Kim and Hong showed that increments in PM_10_ and NO_2_ levels were significantly associated with changes in blood glucose levels among the elderly population in Seoul, Korea [5]. This study was performed on a panel study upon elderly subjects in a district. Few studies have evaluated some diabetes-related blood markers that can be used to determine the presence of diabetes based on a general population.

We explored the association between short- and mid-term exposure to ambient air pollution and blood markers of DM with a specific focus on susceptibility factors, such as sex, age, and the presence of DM, using the Korea National Health and Nutrition Examination Survey (KNHANES).

## Methods

### Study population

We used the data from the KNHANES, which is an annual nationwide survey conducted by the Korea Centers for Disease Control and Prevention since 1998 [25]. It is a cross-sectional survey composed of a health interview, physical examination, and nutrition survey applied to a nationally representative population [26]. This survey provides representative and reliable statistical data on 23 households from 192 national sampling units on the level of health, health behavior, and food and nutritional intake in the Republic of Korea (South Korea). The sampling framework of the survey uses the latest population and housing survey data available at the time of sample design so that a probability sample can be extracted for those over one year of age living in South Korea. Two-stage stratified sampling was applied to select the primary and secondary sampling units. The KNHANES also developed a method for quality control and diagnostic medical examinations to ensure the accuracy of blood test results and conducts internal and external quality control evaluations each year. A total of 7279 in males and 8797 in females participated in the KNHANES in 2012 and 2013. Among them, we recruited those aged 20 years or older.

The present study used the FBG and HbA1c values obtained after the participants had fasted for 8 h to determine the presence of DM from blood tests. We selected only those who had also responded to the questionnaire among those with data on FBG and HbA1c levels. Participants with FBG levels above 126 mg/dL or taking diabetes medications, who received insulin injections, or who had been diagnosed with DM by a physician were classified as being diabetic. We calculated the body mass index (BMI) using the height and weight measured during the health examination. BMI was defined as body weight (kg) divided by the square of height (meters). During the study period, a total of 10,014 participants were included in the analysis (4267 in male and 5747 in female).

### Definition of covariates

Individual socioeconomic status (SES) was determined based on the participants’ level of education, which was categorized based on the participants’ responses to the items in the questionnaire used in the KNHANES: “below elementary school”, “middle school”, “high school”, and “university and over”. Alcohol consumption status was categorized as “no”, “less than once a week”, and “more than once a week” [27]. Physical activity was classified as “no” if no physical activity was performed and “yes” if at least moderate physical activity was performed once a week. Smoking status was categorized as “never smoked”, “smoked in the past but not currently”, and “currently smoking” [28, 29]. According to BMI, we categorized the participants as “underweight” (BMI < 18.5 kg/m^2^), “normal” (BMI ≥18.5 and < 25.0 kg/m^2^), and “obese” (BMI ≥25.0 kg/m^2^) [30, 31]. The level of FBG (mg/dL) and HbA1c (percentage) per interquartile range (IQR) was adjusted for daily mean temperature, humidity, sex, age, education, alcohol consumption, physical activity, smoking, and obesity in Model 3 (Additional file 1: Table S1 and S2).

### Exposure modeling

Of 192 sampling units in the KNHANES, only half had air pollution data measured at air quality monitoring stations. In order to generate air pollution data for all 192 sampling units, a prediction model with data assimilation using data from the Korean Air Quality Forecasting System (KAQFS) was used.

The modeling configuration of the KAQFS consists of the Weather Research and Forecasting model for meteorological modeling, the Sparse Matrix Operator Kernel Emissions system for emission data processing, and the Community Multiscale Air Quality (CMAQ) model for chemical transport simulation over the East Asia region, focusing on the Korean peninsula. The KAQFS, which has been running and open to the public since 2007 (http://www.kaq.or.kr/), was used to forecast the air quality in terms of PM_10_, PM_2.5_, SO_2_, O_3_, CO, and NO_2_ concentrations across South Korea. The anthropogenic emission data from China and Japan were based on the Multi-resolution Emission Inventory for China (http://www.meicmodel.org) for 2010 and the Regional Emission inventory in Asia for 2008 from the National Institute for Environmental Studies, Japan. For the South Korean region, the Clean Air Policy Support System emission inventory by the National Institute for Environmental Research for 2011 was used. The detailed model configurations and corresponding input data have been described elsewhere [32–36].

The data assimilation using surface measurements in China and South Korea was applied to the KAQFS to enhance model performance. The model predictions using the KAQFS with data assimilation showed better agreement with observations than those without it. Choi et al. showed that the spatial distributions of PM_10_, PM_2.5_, and NO_2_ with data assimilation over China and the Korean peninsula accurately depicted the observed air quality at the monitoring stations [36]. Further details of data assimilation are available in Choi et al.’s study, and these data have also been validated in a previous study [36, 37].

The air quality over the Korean peninsula was assessed according to grid sizes of 9 km × 9 km, with a fine grid size of 3 km × 3 km in the metropolitan areas of Seoul. We used these values to calculate the weighted mean values according to the boundaries of 253 health administrative districts. We estimated the daily concentrations of PM_10_ (unit: μg/m^3^), PM_2.5_ (unit: μg/m^3^), and NO_2_ (unit: parts per billion [ppb]) using the KAQFS data in each region where the KNHANES is conducted. The data were combined by matching the residential address of the participants and the date of the blood test.

### Study design

To assess the susceptibility factors, we stratified the participants into males and females and divided according to age group (< 65 and ≥ 65 years). The changes in FBG and HbA1c levels were then analyzed according to the level of exposure to air pollution. In particular, we observed how the effects of air pollution differed in diabetic and non-diabetic participants. In order to observe changes in FBG levels due to short-term exposure to air pollution, a lag effect from a moving average of 0–10 days before the date of the blood test (0 day [same date of the blood test], 0–1 days, 0–2 days, … 0–10 days) was applied, and the effect of relatively mid-term exposure on HbA1c levels was examined. HbA1c has been widely accepted as the most reliable method for evaluating mid-term blood glucose control in participants with diabetes and reflects the glucose levels in the previous 6–8 weeks as the lifespan of a typical red blood cell is approximately 115 days [38]. HbA1c should not be used for monitoring rapid changes in blood glucose levels [39, 40]. To estimate the HbA1c level changes due to mid-term exposure to air pollution, we calculated the average exposure by applying the moving averages on days 0, 0–10, 0–15, 0–30, 0–45, 0–60, 0–75, 0–90, 0–120, and 0–150 before the blood test. The changes in FBG and HbA1c levels were analyzed by applying the IQR of air pollutants during the study period.

### Statistical analysis

We used the generalized additive model (GAM) to observe the changes in FBG and HbA1c levels with increasing concentrations of air pollutants. The use of GAM enables a non-linear fit for each variable while maintaining the legibility of existing linear models. We used the generalized linear model, an extension of the linear model, in which the dependent variable is continuous. In each model, the air pollutant value was regarded as an independent variable, while the dependent variables were the levels of FBG and HbA1c in consideration of the lag effect. All models were adjusted for sex, age, level of education, alcohol consumption, physical activity, smoking, obesity, and splined function of daily mean temperature and humidity at the time of exposure. All statistical analyses were performed using SAS version 9.4 (SAS Institute Inc., Cary, NC, USA) and R version 3.5.3 (https://cran.r-project.org/bin/windows/base/old/3.5.3/). The level of statistical significance was set at *p* = 0.05, and the 95% confidence intervals (CIs) were estimated for the point estimates.

## Results

A total of 4267 in males and 5747 in females were included in the analyses. According to the criteria for diagnosing diabetes, 13.1% of males and 9.6% of females had diabetes (Table 1).

**Table 1:** Demographic characteristics of the study participants by sex in the Korea National Health and Nutrition Examination Survey (2012–2013)

Variables	Male		Female		Total		***p***-value^**†**^
	(*n* = 4267)		(*n* = 5747)		(*n* = 10,014)		
	N	%	N	%	N	%	
Diabetes^a^							< 0.0001
No	3710	86.9	5194	90.4	8904	88.9	
Yes	557	13.1	553	9.6	1110	11.1	
Age group							0.565
< 65 years	3294	77.2	4495	78.2	7789	77.8	
≥65 years	973	22.8	1252	21.8	2225	22.2	
Education level							< 0.0001
Elementary school	694	16.3	1651	28.8	2345	23.4	
Middle school	459	10.8	589	10.2	1048	10.5	
High school	1577	36.9	1858	32.3	3435	34.3	
University and over	1537	36.0	1649	28.7	3186	31.8	
Alcohol consumption							< 0.0001
No	724	17.0	2151	37.4	2875	28.7	
Less than once a week	1994	46.7	3086	53.7	5080	50.7	
More than once a week	1549	36.3	510	8.9	2059	20.6	
Physical activity							< 0.0001
No	2338	54.7	2201	38.3	4539	45.3	
Yes	1929	45.2	3546	61.7	5475	54.7	
Smoking							< 0.0001
Never smoked	873	20.5	5160	89.8	6033	60.2	
Past	1703	39.9	265	4.6	1968	19.7	
Current	1691	39.6	322	5.6	2013	20.1	
Obesity							< 0.0001
Underweight (BMI: < 18.5 kg/m^2^)	96	2.2	301	5.2	397	63.5	
Normal (BMI: ≥18.5 and < 25.0 kg/m^2^)	2601	61.0	3753	65.3	6354	4.0	
Obesity (BMI: ≥25.0 kg/m^2^)	1570	36.8	1693	29.5	3263	32.5	

^a^*p*-values were obtained by comparing the groups using the chi-square test or Fisher’s exact test. ^‡^ Participants with FBG levels above 126 mg/dL or taking diabetes medications, who received insulin injections, or who had been diagnosed with DM by a physician were classified as being diabetic

The mean levels of exposure to PM_10_ and PM_2.5_ were 42.1 and 32.1 μg/m^3^, respectively, twice as high as the World Health Organization’s annual recommendation (20 and 10 μg/m^3^, respectively). The mean level of exposure to NO_2_ was 23.5 ppb. The IQRs of PM_10_, PM_2.5_, and NO_2_ were 23.5 μg/m^3^, 19.4 μg/m^3^, and 16.7 ppb, respectively (Table 2). During the study period, the daily mean temperature was 9.8 °C, while the relative humidity was 58.0%.

**Table 2:** Exposure to air pollutants and meteorological indexes during the study period

	Mean	SD	Min	Percentile			Max	IQR
				25th	50th	75th		
Daily exposures								
PM_10_ (μg/m^3^)	42.1	22.0	3.6	27.2	37.0	50.7	152.9	23.5
PM_2.5_ (μg/m^3^)	32.1	17.7	2.8	19.8	28.4	39.2	147.8	19.4
NO_2_ (ppb)	23.5	13.7	0.8	13.8	20.4	30.5	76.2	16.7
Temperature (°C)	9.8	10.5	−19.5	1.3	9.8	19.7	28.8	18.3
Humidity (%)	58.0	18.5	12.3	44.4	57.7	73.1	95.1	28.7

The concentration of PM_10_, PM_2.5_, and NO_2_ was estimated using the Community Multiscale Air Quality model. SD, standard deviation; IQR, interquartile range; PM_10_, particulate matter < 10 μm; PM_2.5_, particulate matter < 2.5 μm; NO_2_, nitrogen dioxide; ppb, parts per billion.

Predicted values of the CMAQ model using data assimilation was compared with the observations at the Korean Air Quality monitoring station (AQMS) as was shown in Fig. S1 (Additional file 2). PM_10_ and NO_2_ were measured at all AQMSs, but the PM_2.5_ was only monitored in Seoul metropolitan city during 2012 and 2013. The results of the cross-validation for daily mean PM_10_, PM_2.5_ and NO_2_ are summarized in Fig. S2 (Additional file 2). They showed that CMAQ model could reproduce observations for entire region in South Korea with R^2^-square was 0.78 for PM_10_, 0.59 for PM_2.5_ and 0.86 for NO_2_ respectively.

**Fig. 1:**
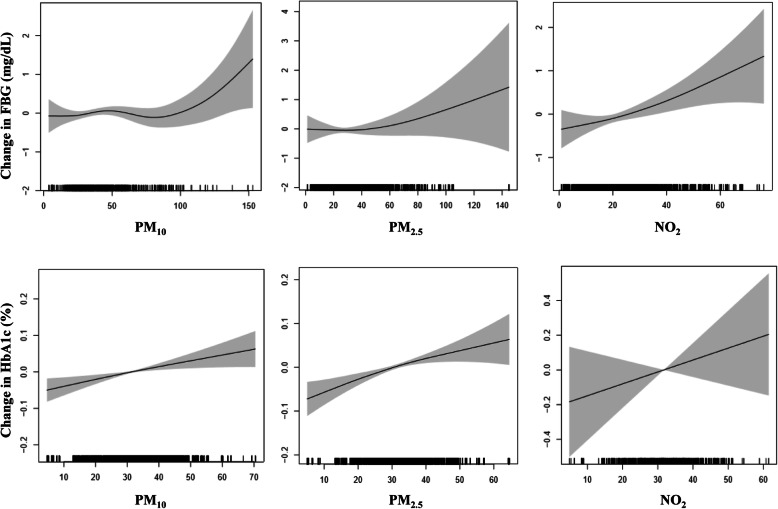
Change in fasting blood glucose (mg/dL)^a^ and hemoglobin A1c (HbA1c, %)^a^ levels per unit increment of air pollutants in the diabetic group. ^a^Adjusted for sex, age, education level, smoking, alcohol consumption, physical activity, obesity, daily mean temperature, and humidity. PM_10_, particulate matter < 10 μm; PM_2.5_, particulate matter < 2.5 μm; NO_2_, nitrogen dioxide; ppb, parts per billion. **a** Changes in fasting blood glucose (FBG) levels at a moving average of 0–6 days for PM_10_ and PM_2.5_, and 0–7 days for NO_2_. **b** Changes in HbA1c level at a moving average of 0–60 days for each pollutant

We examined the FBG and HbA1c levels of the study participants to determine the lag effect. Figure 1 shows the changes in FBG levels per 1unit increment of PM_10_ and PM_2.5_ with a lag of up to 6 days and NO_2_ with a lag of up to 7 days. GAM analysis demonstrated the dose-response relationship between air quality and glucose metabolites. An increase in the FBG level was evident at PM_10_ of above 75 μg/m^3^ and PM_2.5_ of above 30 μg/m^3^. The relationship was more linear for NO_2_. Considering that the level of HbA1c represents blood glucose levels 6 to 8 weeks before the test, we observed changes in the moving average concentrations of all pollutants at 0–60 days. As the concentration of air pollutants increased, the level of HbA1c also increased, with PM_10_ and PM_2.5_ showing a clear dose-response effect.

We divided all participants into non-diabetic and diabetic and under 65 years and 65 years or over groups to identify effect modifications. As shown in Table 3, the level of FBG was significantly increased by 0.85 mg/dL (95% CI: 0.23–1.47) per IQR increment of NO_2_ and the level of HbA1c was increased by 0.07% (95% CI: 0.02–0.11) per IQR increment of PM_2.5_ in all participants. The FBG level increased by 0.36 mg/dL (95% CI: 0.11–0.62) per IQR increment of NO_2_ in non-diabetic participants, with the peak increased being 3.32 mg/dL (95% CI: 0.64–6.00) in diabetic individuals; however, the HbA1c levels showed no such significant changes. Conversely, exposure to PM_10_ and PM_2.5_ increased the HbA1c levels in the non-diabetic group by 0.04% (95% CI: 0.02–0.06) and 0.06% (95% CI: 0.03–0.08), respectively. Overall, the daily change in FBG and HbA1c levels were higher in the diabetic participants.

**Table 3:** Estimated changes in fasting blood glucose (mg/dL) and hemoglobin A1c (percentage) levels per interquartile range increment of PM_10_, PM_2.5_, and NO_2_ in all participants according to the presence of diabetes

Pollutant	Fasting blood glucose (mg/dL)^**a**^		HbA1c (percentage points)^**a**^	
	***β*** (95% CI)	***p***-value	***β*** (95% CI)	***p***-value
Total				
PM_10_ (μg/m^3^)	0.22 (−0.31–0.75)	0.419	0.05 (0.00–0.10)	0.075
PM_2.5_ (μg/m^3^)	0.31 (−0.20–0.82)	0.227	0.07 (0.02–0.11)^*^	0.002
NO_2_ (ppb)	0.85 (0.23–1.47)^*^	0.006	0.01 (−0.02–0.02)	0.579
No diabetes				
PM_10_ (μg/m^3^)	0.00 (−0.22–0.22)	0.729	0.04 (0.02–0.06)^*^	*p* < 0.0001
PM_2.5_ (μg/m^3^)	−0.09 (− 0.30–0.12)	0.383	0.06 (0.03–0.08)^*^	*p* < 0.0001
NO_2_ (ppb)	0.36 (0.11–0.62)^*^	0.004	0.01 (−0.01–0.01)	0.618
Diabetes				
PM_10_ (μg/m^3^)	3.28 (0.60–5.97)^*^	0.016	0.16 (−0.08–0.41)	0.194
PM_2.5_ (μg/m^3^)	2.83 (0.27–5.38)^*^	0.030	0.15 (−0.07–0.37)	0.186
NO_2_ (ppb)	3.32 (0.64–6.00)^*^	0.015	0.05 (−0.11–0.21)	0.529

^*^p-value < 0.05. The level of fasting blood glucose (mg/dL) per interquartile range increment at a moving average of 0–6 days in PM_10_ and PM_2.5_ and 0–7 days in NO_2_. The level of HbA1c (percentage) per interquartile range increment at a moving average of 0–60 days for each pollutant. ^a^Model 3, adjusted for sex, age, education level, physical activity, smoking, alcohol consumption, obesity, daily mean temperature, and humidity. CI, confidence interval; PM_10_, particulate matter < 10 μm; PM_2.5_, particulate matter < 2.5 μm; NO_2_, nitrogen dioxide; ppb, parts per billion

In participants aged ≥65 years, exposure to NO_2_ strongly increased the level of FBG (0.39 mg/dL; 95% CI: 0.12–0.66) not only in non-diabetic but also in diabetic individuals (4.92 mg/dL; 95% CI: 1.27–8.57) (Table 4). In all participants, the HbA1c level increased by 0.07% (95% CI: 0.03–0.09) with exposure to PM_10_ and by 0.09% (95% CI: 0.04–0.14) with exposure to PM_2.5_. When classified according to the presence of diabetes, significant associations were noted in the non-diabetic group (0.07% [95% CI: 0.01–0.12] in PM_10_ and 0.08% [95% CI: 0.03–0.14] in PM_2.5_). In the diabetic group, the peak change in HbA1c levels was 0.12% (95% CI: 0.03–0.20) with exposure to PM_2.5._ Analysis of the relative moving average concentration of air pollutants considering its half-life in the human body revealed that, overall, the increase in HbA1c level was greater in the diabetic group than that in the non-diabetic group. At the same time, while mid-term exposure leads to a significant increase in the HbA1c level in the non-diabetic group. However, exposure to NO_2_ did not show any significant results, and the patterns of change in blood glucose markers were similar for PM_10_ and PM_2.5_.

**Table 4:** Estimated changes in fasting blood glucose (mg/dL) and hemoglobin A1c (percentage) level per interquartile range increment of PM_10_, PM_2.5_, and NO_2_ in participants aged ≥65 years according to the presence of diabetes

Pollutant	Fasting blood glucose (mg/dL)^**a**^		HbA1c (percentage points)^**a**^	
	***β*** (95% CI)	***p***-value	***β*** (95% CI)	***p***-value
Total				
PM_10_ (μg/m^3^)	0.76 (−0.18–1.71)	0.112	0.07 (0.03–0.09)^*^	*p* < 0.0001
PM_2.5_ (μg/m^3^)	0.84 (−0.10–1.77)	0.078	0.09 (0.04–0.14)^*^	*p* < 0.0001
NO_2_ (ppb)	1.09 (0.11–2.06)^*^	0.029	0.00 (−0.02–0.01)	0.445
No diabetes				
PM_10_ (μg/m^3^)	0.23 (−0.28–0.74)	0.380	0.07 (0.01–0.12)^*^	0.015
PM_2.5_ (μg/m^3^)	0.36 (−0.15–0.87)	0.169	0.08 (0.03–0.14)^*^	0.001
NO_2_ (ppb)	0.39 (0.12–0.66)^*^	0.004	0.00 (−0.03–0.04)	0.670
Diabetes				
PM_10_ (μg/m^3^)	3.64 (0.69–6.59)^*^	0.015	0.11 (0.02–0.20)^*^	0.021
PM_2.5_ (μg/m^3^)	3.96 (0.06–7.85)^*^	0.046	0.12 (0.03–0.20)^*^	0.016
NO_2_ (ppb)	4.92 (1.27–8.57)^*^	0.008	0.02 (−0.03–0.08)	0.440

^*^p-value < 0.05. The level of fasting blood glucose (mg/dL) per interquartile range increment at a moving average of 0–6 days for PM_10_ and PM_2.5_ and at 0–7 days for NO_2_. The level of HbA1c (percentage points) per interquartile range increment at a moving average of 0–60 days for each pollutant. ^a^Model 3, adjusted for sex, age, education level, physical activity, smoking, alcohol consumption, obesity, and daily mean temperature and humidity. *CI* confidence interval; *PM*_*10*_ particulate matter < 10 μmM, *PM*_*2.5*_, particulate matter < 2.5 μm; *NO*_*2*_ nitrogen dioxide, *ppb* parts per billion

**Fig. 2:**
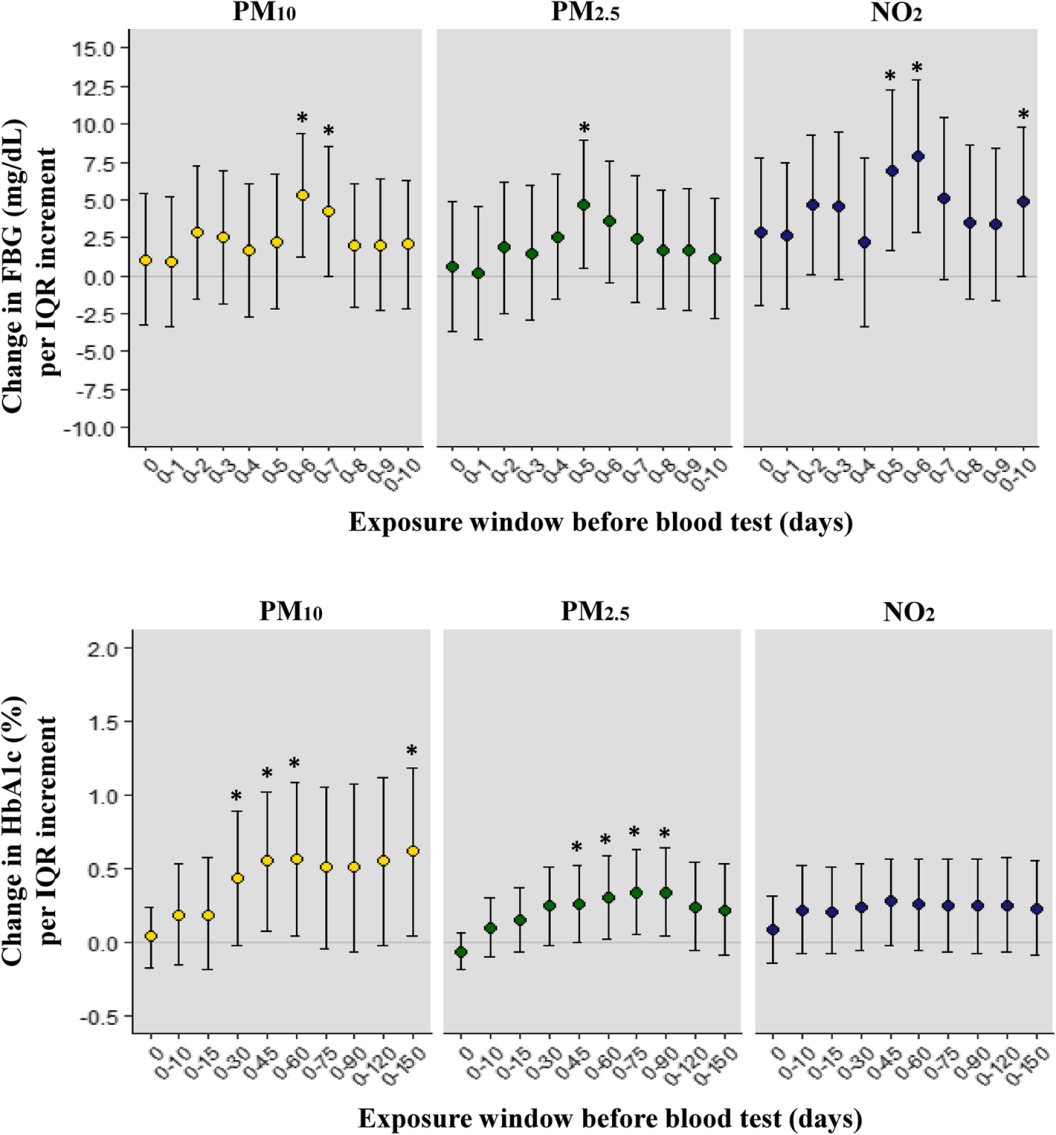
Associations between changes in fasting blood glucose (mg/dL)^a^ and HbA1c (%)^a^ levels per interquartile range increment of PM_10_, PM_2.5_, and NO_2_ in diabetic male aged ≥65 years. ^*^p < 0.05. ^a^Adjusted for age, education level, smoking, alcohol consumption, physical activity, obesity, daily mean temperature, and humidity. PM_10_, particulate matter < 10 μm; PM_2.5_, particulate matter < 2.5 μm; NO_2_, nitrogen dioxide; ppb, parts per billion. 0, date of blood test; 0–15, moving average from days 0 to 15; 0–30, moving average from days 0 to 30; 0–45, moving average from days 0 to 45; 0–60, moving average from days 0 to 60; 0–75, moving average from days 0 to 75; 0–90, moving average from days 0 to 90; 0–120, moving average from days 0 to 120; 0–150, moving average from days 0 to 150

Based on the overall results, we performed a subgroup analysis based on sex and found that FBG levels were the highest in diabetic male aged 65 years and over (5.32 mg/dL [95% CI: 1.22–9.41] for PM_10_ and 4.69 mg/dL [95% CI: 0.48–8.91] for PM_2.5_) with a lag of up to six days and five days, respectively, but did not differ significantly in female (Fig. 2). In particular, according to the level of daily exposure to NO_2_, the highest increase was by 7.83 mg/dL (95% CI: 2.80–12.87) in males with a lag of up to 6 days. Although the HbA1c level did not increase significantly with exposure to NO_2_, it increased with exposure to PM_10_ and PM_2.5_. The highest increase (0.57, 95% CI: 0.04–1.09) occurred for PM_10_ at a moving average of 0–60 days and at 0–75 days and 0–90 days for PM_2.5_ (0.34, 95% CI: 0.04–0.63 and 0.34, 95% CI: 0.05–0.64, respectively).

## Discussion

We explored the effects of short-term exposure to air pollution on changes in FBG levels and mid-term exposure on changes in HbA1c levels considering effect modifiers using the KNHANES data combined with CMAQ modeling to determine the level of exposure to air pollution. The level of exposure was calculated according to the same administrative districts in which the survey had been conducted. The findings obtained from our study indicated that the levels of FBG and HbA1c were more sensitive to exposure to air pollution in males and that the susceptible groups were the elderly with diabetes.

Previous studies showed the association of pathophysiologic pathways and glucose metabolism with air pollution. Inhaled air pollutants induce oxidative stress and inflammation in the lungs and damage other organ systems, including the adipose tissue [41–44]. This inflammation negatively affects the insulin signaling pathways that regulate glucose metabolism, resulting in disruption of blood glucose regulation and an abnormal increase in glucose level. In diabetic participants with high insulin insensitivity, a dramatic increase in FBG and HbA1c levels occurs due to the influx of harmful substances. Based on this evidence, epidemiological studies on the increased prevalence of DM due to exposure to harmful substances in ambient air and changes in biological factors related to diabetes are actively conducted. The findings of the present study were consistent with those of previous studies. Kim and Hong [5] reported that in elderly Koreans with a history of DM, based on IQR increments of PM_10_ and NO_2_, the FBG levels were elevated by 7.74 mg/dL and 9.90 mg/dL, respectively. In comparison, we observed an increase of 8.74 mg/dL per 23.5 μg/m^3^ increment in PM_10_ and 15.32 mg/dL per 16.7 ppb increment of NO_2_ in diabetic male aged 65 years and over in the present study. Although our values were slightly lower, the results were similar to those reported in Kim and Hong’s study. While the sensitivity of the vulnerable groups varied according to the level of air pollution exposure in each country and differences in living environments, we compared the sensitivity with that reported in studies from other countries. Liu et al. reported increase in FBG level of 2.20 mg/dL and HbA1c level of 0.04% with a 19.4 μg/m^3^ increment in PM_2.5_ [17]. Chuang et al. reported an estimated change in HbA1c level of 0.06% per 34 μg/m^3^ increment following short-term exposure to PM_10_ [20]. When the IQR values of our study were applied, the change of HbA1c in Chuang et al.’s study was 0.04%, which was slightly lower than that in our study. In the study by Lucht et al., the same exposure period as in our study was applied but not in the diabetic group [23]. For 91-day exposure, a 4 μg/m^3^ increment in PM_2.5_ elevated the HbA1c level by 0.07%, while a 5.5 μg/m^3^ increment in PM_10_ increased the HbA1c level by 0.04%; no significant changes were observed with exposure to NO_2_, as in our results. In a study in northern France, which accessed long-term exposure to air pollution and HbA1c levels, based on which we converted the exposure level in South Korea, the level of HbA1c increased by 0.52% per 23.5 μg/m^3^ increment of PM_10_, similar to that shown in the current study [45].

The FBG level was altered with short-term exposure, especially in diabetic males aged 65 years or more. This population was sensitive to exposure to PM_10_ and PM_2.5_ but more responsive to NO_2_ exposure, with an increase of 7.83 mg/dL (95% CI: 2.80–12.87) per IQR increment of NO_2_. Considering that HbA1c represents the average blood glucose level over a few months, it revealed significant changes according to exposure at a moving average of 0–45, 60, and 75 days in diabetic males aged 65 years or more, which is consistent with the results of previous studies. Considering that an increase in HbA1c level of 1% corresponds to an average FBG level increase of 35 mg/dL, a change in HbA1c level cannot be considered small. This observation also suggests that both short-term and mid-term exposure can significantly increase the risk of diabetes. Although most significant results were shown in males aged over 65 years in the present study, the risk of DM in females cannot be neglected. The sexual difference in the health effects of air pollutants may be attributable to hormonal characteristics in addition to the differences in the frequency of exposure to pollutants over their lifetime. In a previous study, middle-aged males with higher serum testosterone levels and sex hormone-binding globulin had high insulin sensitivity, which was independent of baseline insulin levels, body weight, and fat [46]. Lower than normal serum testosterone levels caused by hypogonadism increased the risk of developing glucose metabolic disorders and metabolic syndrome [47]. Previous studies reported that the serum testosterone levels in nearly 80% of men, age 60 to 80 years, decrease rapidly every ten years until such time that they become testosterone deficient [48, 49]. The effects of these factors could be more pronounced owing to synergistic and cumulative effects in an elderly male with relatively high exposure to hazardous substances due to ambient air pollution. Results of the present study confirm this association based on the observed increased effect of air pollution exposure on diabetes-related blood markers in vulnerable groups in contrast to that in non-diabetic individuals. The inhalation of toxic substances in individuals with compromised metabolic function due to chronic diseases may disrupt glucose homeostasis by weakening the immune system and further decreasing metabolic function.

We observed changes in diabetes-related parameters caused by exposure to air pollution in consideration of effect modifiers; such effects were also noted in the absence of diabetes. Peng et al. and Lucht et al. observed significantly elevated FBG levels in non-diabetic participants; they concluded that the effects of air pollution might be relatively minor in diabetic individuals who consume a well-balanced diet, who perform physical activities, and who receive insulin or hypoglycemic agents [18, 23]. These findings suggest that more attention should be paid in managing the susceptible groups in a community rather than for early screening of the entire population. The trend of changes in FBG and HbA1c levels caused by air pollution were not similar. In particular, unlike FBG, the HbA1c level did not show any significant effect of exposure to NO_2_ and was more affected by fine particulate matter, as shown in some previous studies [23, 50].

Most studies on the health effects of air pollution in South Korea were conducted using atmospheric environmental data measured at domestic monitoring sites, which are mostly installed in metropolitan or industrial areas. In terms of health administration units, only half of the units have air monitoring posts. There is a limitation in identifying health effects based on health index generated at the local level every year. Generally, airborne exposure data collected from domestic monitoring sites are lower in value than the actual exposure levels in areas without monitoring sites [51]. To overcome these problems, we used the KAQFS and CMAQ data to match the survey areas of the KNHANES. Finally, the currently available KAQFS and CMAQ data only show the results within two years; hence, long-term data were not available for this study. The level of air pollution varies each year, and there is a need to understand the longer-term variations to obtain more accurate results. In addition, previous studies reported that the traffic-related air pollution increase the risk of DM [9, 11, 16]. It is necessary to build a model of air pollutants emitted from major roads such as the Land Use Regression model to present an increased risk of diabetic-related indicators following exposure to the ambient air pollution.

The greatest strength of our analysis is owing to the nationwide survey which is well standardized with good quality control.. In Korea, studies on diabetes-related metabolism and exposure to ambient air pollution have not been conducted except for that conducted by Kim and Hong [5]. Our study was based on a national survey of the population, allowing a comparison with other countries. In particular, our analysis was based on the results of blood tests obtained from examinations performed during the KNHANES, a typical nationwide survey with reasonable quality control, which is performed annually through a systematic quality control system [25].

Second, we generated air pollution exposure data for health administrative districts that were surveyed using modeling data and matched these data to the residential address of the participants and the exact date of the blood test. The date of the blood test was used to identify changes in the FBG level due to short-term exposure to air pollution and that in HbA1c level due to mid-term exposure. Our findings provide a potential explanation for why diabetic individuals are at a higher risk for DM due to acute exposure to air pollution, although the effects on glucose metabolism of short- and mid-term exposure to air pollution may differ from chronic effects.

Third, we estimated the effects of air pollution on diabetes-related indicators in terms of effect modifiers using KNHANES data extracted based on sample design guidelines. Although the disruption in glucose metabolism owing to ambient air pollution has been reported worldwide, these effects are observed in susceptible subpopulations more rather than in the entire population. Based on previous studies, we stratified all participants according to effect modifiers to define sensitive group and compared the effects on each vulnerable group to estimate the representative values. The findings of the current study suggest that the groups susceptible to DM due to air pollution should be clearly defined and that approaches for the prevention of chronic diseases should be developed.

We found that there is a strong association between the short-term exposure to air pollution and diabetes-related factors in adults over the age of 20. This suggests that this continuous exposure affects the metabolism of insulin, including FBG and HbA1c, and may be a risk factor for the development of metabolic diseases such as glucose intolerance or type 2 diabetes later in life. In addition, risk factors for diabetes metabolism, including insulin resistance, are rarely perceived as harmful in early life, but over time, such as long-term exposure, it can be tracked the risk of DM and lead to higher risks [52, 53]. Therefore, based on our findings, it suggests that scientific evidence should be established based on studies on the risks of metabolic-related factors from short-term and long-term exposure to air pollution in South Korea.

The rapid industrialization of South Korea and surrounding East Asian areas has led to increased air pollution, which is a major environmental problem. Specifically, there is concern regarding the adverse health effects of long-term exposure to air pollution. In the present study, we quantitatively assessed the health effects at the local level by combining atmospheric data and health behavior indices and biomarkers in the KNHANES performed in health administrative districts annually. Considering the increasing incidence of diabetes as a chronic disease and an increase in air pollution in South Korea, policy changes to reduce the health effects caused by exposure to air pollution in the elderly population are necessary for adequate health and environment management. Evaluation of the risk of diabetes and chronic diseases due to exposure to air pollution in susceptible populations in advance can be used to develop customized preventive programs at the local level.

## Conclusion

We found an increased risk of elevated blood glucose levels with short- and mid-term exposure to air pollution, which was more prominent among diabetic males aged ≥65 years in South Korea. These results suggest that policy changes should be made for adequate health and environment management in order to reduce the health effects of air pollution on elderly people and the increasing prevalence of diabetes, taking into account the severity of chronic diseases and air pollution.

## Supplementary information

**Additional file 1: Table S1.** Estimated the Akaike Information Criterion (AIC) for model selection with covariates. **Table S2.** Associations between ambient air pollution and the level of fasting blood glucose level (mg/dL) and hemoglobin A1c (percentage points) per interquartile range of PM_10_, PM_2.5_, and NO_2. (DOCX 17 kb)_

**Additional file 2: Figure S1.** The location of the Korean Air Quality monitoring stations (AQMS) in South Korea with highlighted box of Seoul Metropolis. **Figure S2.** The results of cross-validation for daily mean concentration of PM10, PM2.5, and NO2 in South Korea during 2012 and 2013. x-axis: observed values. y-axis: predicted values.

## Data Availability

Not applicable.

## Abbreviations

AQMS: Air Quality monitoring station
BMI: Body mass index
CI: Confidence interval
CMAQ: Community Multiscale Air Quality
DM: Diabetes mellitus
FBG: Fasting blood glucose
GAM: Generalized additive model
HbA1c: Hemoglobin A1c
IQR: Interquartile range
KNHANES: Korea National Health and Nutrition Examination Survey
KAQFS: Korean Air Quality Forecasting System
NO_2_: Nitrogen dioxide
PPB: Parts per billion
PM_10_: Particulate matter < 10 μm
PM_2.5_: Particulate matter < 2.5 μm
SES: Socioeconomic status
