# Supplementary material for: Impacts of ambient air pollution on glucose metabolism in Korean adults: a Korea National Health and Nutrition Examination Survey study
Source: Environ Health. 2020 Jun 17;19:70. doi: 10.1186/s12940-020-00623-9 (PMC7302244; doi:10.1186/s12940-020-00623-9)
Supplement: Supplementary file 1 — Additional file 1: Table S1. Estimated the Akaike Information Criterion (AIC) for model selection with covariates. Table S2. Associations between ambient air pollution and the level of fasting blood glucose level (mg/dL) and hemoglobin A1c (percentage points) per interquartile range of PM10, PM2.5, and NO2. (DOCX 17 kb) [file 12940_2020_623_MOESM1_ESM.docx]

**Additional file**

**Table S1.** Estimated the Akaike Information Criterion (AIC) for model selection with covariates

| **Model** | **Covariates** | **AIC^†^** |
| --- | --- | --- |
| Model 1 | sex+age+alcohol consumption+physical activity+smoking | 89577.91 |
| Model 2 | Model 1+education level | 89444.18 |
| Model 3 (main) | Model 2+obesity | 89240.41 |

^†^: Estimated using generalized additive model. AIC, Akaike Information Criterion.

**Table S2.** Associations between ambient air pollution and the level of fasting blood glucose level (mg/dL) and hemoglobin A1c (percentage points) per interquartile range of PM_10_, PM_2.5_, and NO_2_

| ***β* (95% CI)** | **Air pollutants** | | |
| --- | --- | --- | --- |
|  | **PM_10_ (μg/m^3^)** | **PM_2.5_ (μg/m^3^)** | **NO_2_ (ppb)** |
| FBG (mg/dL) |  | | |
| *β_0_* | 0.49 (0.01–0.97)^*^ | 0.44 (−0.03–0.90) | 1.73 (0.52–2.94)^*^ |
| *β_1_* | 0.40 (−0.07–0.87) | 0.26 (−0.22–0.74) | 1.70 (0.56–2.84)^*^ |
| *β_2_* | 0.22 (−0.31–0.75) | 0.31 (−0.20–0.82) | 0.85 (0.23–1.47)^*^ |
| HbA1c (percentage points) |  | | |
| *β_0_* | 0.18 (0.00–0.36) | 0.12 (−0.04–0.28) | 0.05 (−0.09–0.19) |
| *β_1_* | 0.05 (0.02–0.09)^*^ | 0.06 (0.02–0.09)^*^ | −0.03 (−0.05–0.00) |
| *β_2_* | 0.05 (0.00–0.10) | 0.07 (0.02–0.11)^*^ | 0.01 (−0.02–0.02) |

^*^*p*-value <0.05. The level of fasting blood glucose (mg/dL) per interquartile range increment at moving average of 0–6 days in PM_10_ and PM_2.5_, 0–7 days in NO_2_. The level of HbA1c (percentage points) per interquartile range increment at moving average of 0–60 days for each pollutant. *β_0_*: no adjustment. *β_1_*: *β_0_* + daily mean temperature and humidity. *β_2_*: *β_1_* + sex, age, education level, alcohol consumption, physical activity, smoking, obesity (as in model 3 [main]). CI, confidence interval; PM_10_, particulate matter <10 μm; PM_2.5_, particulate matter <2.5 μm; NO_2_, nitrogen dioxide; ppb, parts per billion.
