# Supplementary figures and images for: Impacts of ambient air pollution on glucose metabolism in Korean adults: a Korea National Health and Nutrition Examination Survey study
Source: Environ Health. 2020 Jun 17;19:70. doi: 10.1186/s12940-020-00623-9 (PMC7302244; doi:10.1186/s12940-020-00623-9)

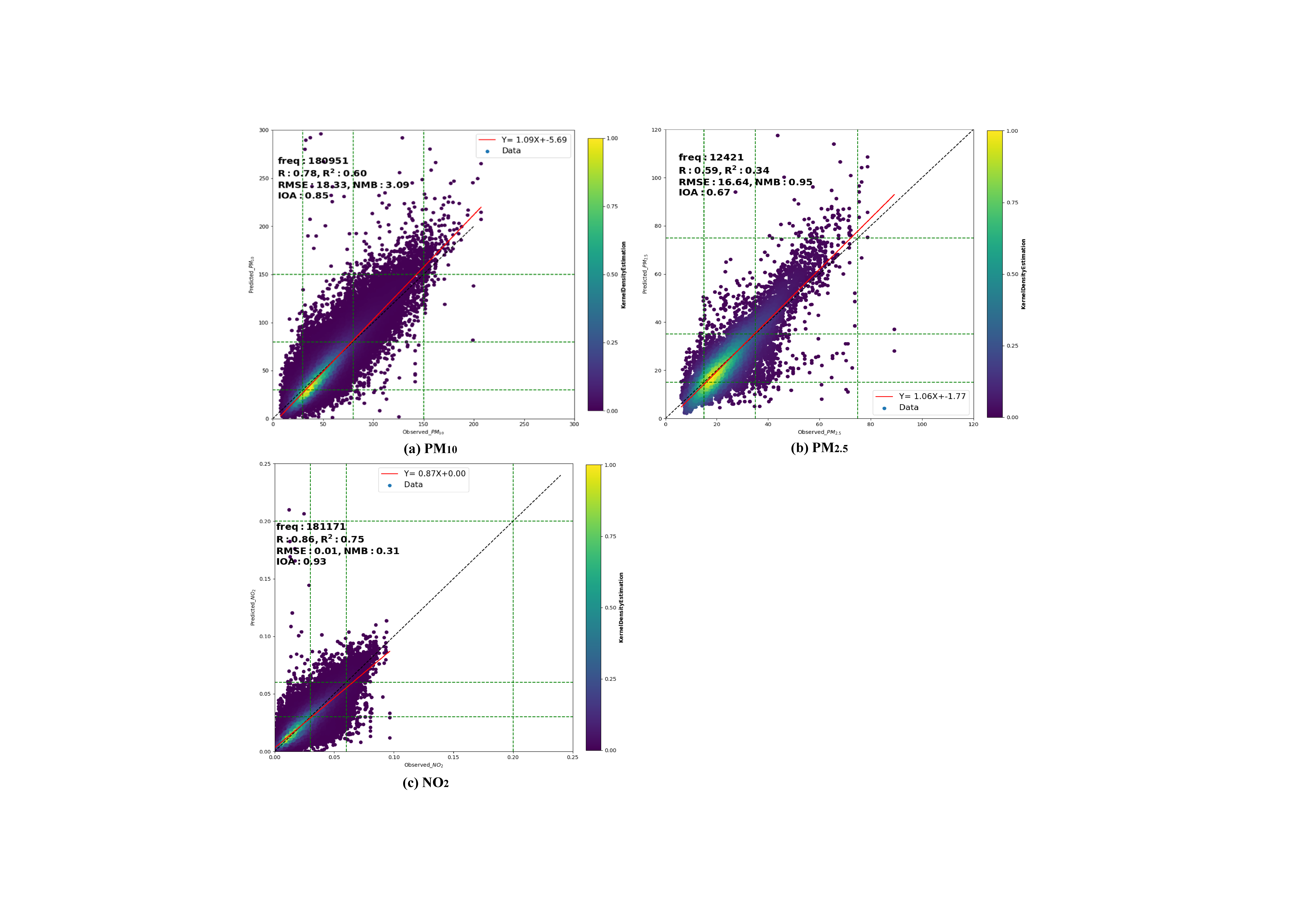

Supplement: Supplementary file 2 — Additional file 2: Figure S1. The location of the Korean Air Quality monitoring stations (AQMS) in South Korea with highlighted box of Seoul Metropolis. Figure S2. The results of cross-validation for daily mean concentration of PM10, PM2.5, and NO2 in South Korea during 2012 and 2013. x-axis: observed values. y-axis: predicted values. [file 12940_2020_623_MOESM2_ESM.zip › Figure S2R1.tiff]
